# Development and application of the ocular immune-mediated inflammatory diseases ontology enhanced with synonyms from online patient support forum conversation

**DOI:** 10.1016/j.compbiomed.2021.104542

**Published:** 2021-08

**Authors:** Samantha C. Pendleton, Karin Slater, Andreas Karwath, Rose M. Gilbert, Nicola Davis, Konrad Pesudovs, Xiaoxuan Liu, Alastair K. Denniston, Georgios V. Gkoutos, Tasanee Braithwaite

**Affiliations:** a Institute of Cancer and Genomic Sciences, University of Birmingham, UK; b University Hospitals Birmingham NHS Foundation Trust, UK; c Health Data Research, UK; d Moorfields Eye Hospital NHS Foundation Trust, London, UK; e Institute of Ophthalmology, University College London, UK; f Olivia's Vision, Southampton Buildings, London, UK; g School of Optometry and Vision Science, University of New South Wales, Australia; h Institute of Inflammation and Ageing, University of Birmingham, UK; i Institute of Applied Health Research, University of Birmingham, UK; j The Medical Eye Unit, St Thomas' Hospital NHS Foundation Trust, London, UK

**Keywords:** Uveitis, Ontology, Inflammation, Patient voice, Sentiment

## Abstract

**Background:**

Unstructured text created by patients represents a rich, but relatively inaccessible resource for advancing patient-centred care. This study aimed to develop an ontology for ocular immune-mediated inflammatory diseases (OcIMIDo), as a tool to facilitate data extraction and analysis, illustrating its application to online patient support forum data.

**Methods:**

We developed OcIMIDo using clinical guidelines, domain expertise, and cross-references to classes from other biomedical ontologies. We developed an approach to add patient-preferred synonyms text-mined from oliviasvision.org online forum, using statistical ranking. We validated the approach with split-sampling and comparison to manual extraction. Using OcIMIDo, we then explored the frequency of OcIMIDo classes and synonyms, and their potential association with natural language sentiment expressed in each online forum post.

**Findings:**

OcIMIDo (version 1.2) includes 661 classes, describing anatomy, clinical phenotype, disease activity status, complications, investigations, interventions and functional impacts. It contains 1661 relationships and axioms, 2851 annotations, including 1131 database cross-references, and 187 patient-preferred synonyms. To illustrate OcIMIDo's potential applications, we explored 9031 forum posts, revealing frequent mention of different clinical phenotypes, treatments, and complications. Language sentiment analysis of each post was generally positive (median 0.12, IQR 0.01–0.24). In multivariable logistic regression, the odds of a post expressing negative sentiment were significantly associated with first posts as compared to replies (OR 3.3, 95% CI 2.8 to 3.9, p < 0.001).

**Conclusion:**

We report the development and validation of a new ontology for inflammatory eye diseases, which includes patient-preferred synonyms, and can be used to explore unstructured patient or physician-reported text data, with many potential applications.

## Highlights

• Here we present OcIMIDo, the first ontology of its kind in ophthalmology.
• We developed the ontology using domain expertise and clinical guidelines.
• Novel synonym extraction method with tf-idf to capture patient voice.
• Facilitates analysis of unstructured text relating to ocular inflammatory diseases.

## Graphical abstract

**Figure:**
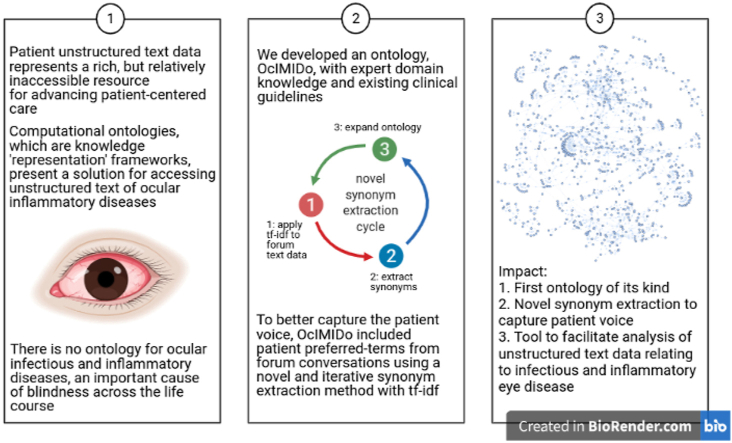

## 1 Introduction

There is growing recognition of the discordance between patient and clinician priorities and perspectives, and the vital importance of integrating the ‘patient voice’ into research and clinical practice [1]. Online patient-reported social media posts, tweets and blogs add a new dimension to the data landscape, and represent a rich, underutilised resource for advancing patient-centred care [1]. However, unstructured text data, and especially text using patient-preferred phrases and terms, rather than formal clinical vocabulary, are relatively inaccessible to extraction and analysis.

Ontologies have been proposed, and demonstrated with great success, to address the challenge presented by unstructured text data [2,3]. Ontologies serve as a computational knowledge representation framework, describing the semantics of biomedical concepts (e.g. clinical phenotypes, clinical signs, complications, investigations, and treatments). Within ontologies these concepts are standardised, and their hierarchy and logical relations facilitate data integration and knowledge sharing [4,5]. In the context of text data, the labels of ontology classes and relations enable access to data or text tagged by them; the classes or relations associated with these labels can be employed to identify potential associations within text descriptions. For this purpose, multiple powerful computational tools have been developed within the biomedical research domain. Used alongside natural language processing (NLP) tools, ontologies facilitate simultaneous, systematic search, tagging and integration of unstructured text data records for all the items (‘classes’) they contain [6]. By encoding specific types of relationships between classes, Machine Learning (ML) and predictive modelling applications can be facilitated [6]. Ontology-guided ML approaches have, in some cases, been shown to achieve better performance than those that do not [7].

### 1.1 Literature review and objectives

There are hundreds of biomedical ontologies, several in widespread use (see Supplementary Table 3) [6,[8], [9], [10], [11], [12], [13], [14], [15]]. Within the medical domain, there is a long history of efforts to systematically represent knowledge across biomedicine (for example medications, procedures, primary and secondary health records etc). Some notable examples include the Unified Medical Language System (UMLS) [16], The International Classification of Diseases (ICD) codes [17] the UK Read codes [18] and the Systematized Nomenclature of Medicine-Clinical Terms (SNOMED CT) [19]. Although, typically, medical terminologies were not originally designed and intended to be used as ontologies, they are hierarchically structured, and have recently adopted formal descriptions and axioms that cater to ontological operations and applications. While each of these ontologies include some classes relevant to a given disease area, rarely they comprehensively define all the important clinical concepts needed for meaningful application in relation to a particular disease and its presentation, investigation, diagnosis, management and impacts. Additionally, recent research has identified an unmet need for biomedical ontologies to also capture synonyms and abbreviations frequently used by patients and physicians [20]. There is a lack of previous research exploring curation of lay synonyms from patient-generated text although one study used word2vec for synonym extraction from a Wikipedia corpus [21].

### 1.2 Aims

Inflammatory eye diseases, including the clinical phenotypes of uveitis, scleritis and optic neuritis, is important because it is sight-threatening, adversely impact quality of life, typically are present throughout the life course, have associations with multi-organ immune-mediated inflammatory diseases of infectious and autoimmune aetiology [22,23] and often require systemic immunosuppressive or immunomodulatory therapy [24].

The primary objective of this study was to report the development of a novel ontology that includes patient preferred terms, the ocular immune-mediated inflammatory diseases ontology (OcIMIDo). Secondary objectives were the application of the ontology to unstructured free text data in two online peer support fora for patients with inflammatory eye disease and their carers. First, we explored frequency of mention of ontology classes (e.g. anterior uveitis, rheumatoid arthritis, or use of methotrexate) in these fora using OcIMIDo and NLP approaches. Second, we performed an exploratory analysis for associations between the OcIMIDo classes and natural language sentiment analysis score at the level of an individual post, using two different sentiment analysis tools, to seek preliminary insight into the psychological impact of disease, medical therapies, and complications. Here we report the successful development, validation and application of OcIMIDo.

## 2 Methods

The University of Birmingham Ethics Committee determined that approval was not required for this study (ERN_20–0047). The research adhered to the tenets of the Declaration of Helsinki.

### 2.1 Development of a new ontology: OcIMIDo

Using Protégé, an open source ontology editor [25], we created OcIMIDo in the Web Ontology Language (OWL) [26]. Our initial foundation classes were extracted and extended by a clinician specialising in inflammatory eye disease, from The Royal College of Ophthalmologists Clinical Dataset (RCOphth) expert consensus document for uveitis [27]. We added additional classes from five widely used biomedical ontologies, on 9th July 2019, via the Ontology Lookup Service [28]. These were the Disease Ontology (DOID) [9], the Human Phenotype Ontology (HPO) [[10], [11], [12]], the Orphanet Rare Disease Ontology (ORDO) [12,13], the Phenotype and Trait Ontology (PATO) [6], and the Uber-anatomy Ontology (UBERON) [15]. We added their term identifiers as cross-reference annotations. We excluded classes that were not relevant to ocular inflammatory diseases (e.g. iris nevus [HP:0011525]). We added relationships using the Relation Ontology [14]. Axioms were automatically populated from the inferred relations within the ontology structure, to include both equivalent class axioms (e.g. high intraocular pressure “Equivalent To” ocular hypertension), and disjointness axioms (e.g. anterior chamber cells grade 0 “Disjoint With” anterior chamber cells grade 1+). We added cross-references between classes and relevant SNOMED-CT [19], ICD-10 [17], and Read Code terms [18], where these were available. Ontology concepts are fully defined in Supplementary Table 4.

### 2.2 Extraction of data from online patient fora

We obtained permission to download and extract text from public online support fora of the UK-based “Olivia's Vision” (OV) charity [oliviasvision.org], and the USA-based, “The Ocular Immunology and Uveitis Foundation” (UVE) organization [uveitis.org]. We downloaded OV on 14 March 2019 and UVE on 6 October 2019, and parsed the fora text data with Python [29], separately identifying threads and their posts, whilst maintaining the anonymity of users.

### 2.3 Patient-preferred synonyms

To identify patient-preferred terms in the OV forum and relate them to clinical terms in OcIMIDo, we used a novel NLP-guided curation technique. Treating each thread as a unique observation, we used equal frequency binning to divide the threads into three size classes (based on the number of posts each contained). To generate a stratified test set, we extracted a random 20% of threads for the test set and used the remaining 80% as the training set. Using the Natural Language Toolkit (NLTK) [30], with each thread in the training set, we cleaned (pre-processed) each post into the same format: lowercase, removal of “stop words” (i.e. words that carry little meaning: “the”, “thanks”, “xx”), and stemming all words to standardise their format across threads (e.g. “pained”, “painful”, and “pains” all became “pain”).

To extract all informative terms from this forum, we proceeded in an iterative way: we added patient-preferred synonyms text-mined from the OV forum, using statistical ranking with the text feature extraction function, “term frequency–inverse document frequency” (tf-idf) in Scikit-learn [31]. The tf-idf statistic is a numerical statistical technique which measures the information in a document and balances it based on size. We applied tf-idf on all documents in the training set, resulting in a word list where higher scores represented more informative words. A domain expert (TB) manually reviewed words scoring above 1.0 to identify meaningful synonyms and classes for inflammatory and infectious eye disease, which we added to OcIMIDo. At the end of this each round of tf-idf, we removed the added synonyms from the training set and re-ran the tf-idf analysis to reweigh remaining words. With the reweighted set, we again curated words identified to be meaningful synonyms and classes, and added these to the ontology. We repeated this process five times and then at the sixth round we identified no additional informative terms. We manually consolidated misspellings into correctly spelled terms during evaluation of tf-idf metrics at each stage.

### 2.4 Validation

First, we performed a tf-idf analysis on the OV test set and manually compared informative words in the training and testing sets to identify any missed classes or synonyms, timing this activity. Second, we performed a tf-idf analysis on the USA-based UVE forum to identify any missed classes or synonyms. Third, two clinicians (RG and XL), not involved in developing the ontology, independently spent a timed 60-min period manually highlighting as many words of relevance to ocular IMID as possible. We compared the number of items identified to those identified by the tf-idf method. We explored differences, including time taken, between manual and tf-idf approaches, and extrapolated to obtain an estimate of the time it would take to manually identify words for inclusion in the ontology.

### 2.5 Using OcIMIDo to explore unstructured text data

We explored the posts across the OV test set using the Stanford CoreNLP [32] suite with the RegexNER annotator to tag named entities in the text. RegexNER added the concept identifiers (e.g. “blurred vision” and it's synonym “blurry” had the same identifier [OCIMIDO:00141]). We observed the frequency of classes (or their synonyms) across the training and testing posts. We repeated this approach to explore and tag the USA-based UVE forum.

### 2.6 Application of OcIMIDo to sentiment analysis

We combined text data from the two fora, OV and UVE. Using TextBlob [33] and VADER [34], we obtained a natural language sentiment score for each post. Both are open source tools for sentiment analysis that use a lexicon-based method to estimate sentiment. An advantage of these tools is that they do not require any training data as they are pretrained models, developed using NLTK [30], thus avoiding the time-consuming task of manually labelling data. Both have been widely used in sentiment analysis of online public free text data, such as research looking into Twitter posts for discussions about chemotherapy [35,36].

We performed exploratory statistical analyses using standard statistical software, Stata (release 13.1) [37]. We sorted the data for each forum by sentiment score, for both TextBlob and VADER independently, and removed 50% from each, retaining only the most positive and most negative 25% of scores. We combined this data, and explored single and then multiple logistic regression models for the odds of a score in the most negative 25th percentile, by a range of clinically relevant potential predictor variables (e.g. different inflammatory subtypes, systemic disease associations, ocular complications, and treatments). We fitted a full exploratory multivariable logistic regression model containing variables with a global p-value of < 0.1 in single variable analysis. We identified the most parsimonious model, which had the smaller value of the Akaike's Information Criterion (AIC) and the Bayesian Information Criterion (BIC), using a stepwise backward elimination approach. We measured the discriminative performance of each model using the area under the receiver operating characteristic curve (AUC). We obtained global p-values, using the likelihood ratio test (LRT). We took a p-value of 0.05 or lower to be statistically significant.

## 3 Results

### 3.1 OcIMIDo

**Fig. 1:**
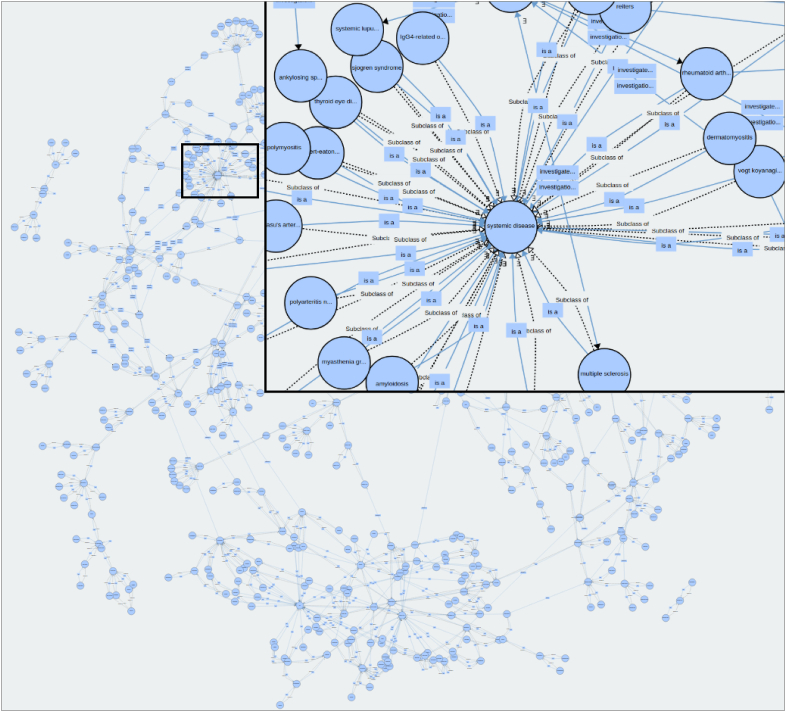
Graphical representation of the ocular immune-mediated inflammatory diseases ontology, with a close-up of systemic, non-infectious disease associations. Each node is a class (e.g. “multiple sclerosis”) and each edge is a relationship, with dotted lines defining “subclass of” and solid lines defining constructed relationships, labelled with boxes (e.g. “investigated by”).

OcIMIDo is open source and freely available via GitHub, accessible at https://github.com/sap218/ocimido. We followed the MIRO guidelines for ontology reporting [38]. OcIMIDo (version 1.2) contained 661 classes, with 210 classes extracted from the foundation document [27], and a total of 1661 relationships (including axioms) (see Fig. 1 created with WebVOWL [39], and Table 1). There were 2851 annotations: 187 of these were synonyms from OV (e.g. generic, proprietary drug names, and commonly used abbreviations) and 1131 were cross-references (700 to medical ontologies, of which 398 were to SNOMED-CT).

**Table 1:** Examples of constructed relationship types contained in OcIMIDo.

Relationship	Count	Cross-reference	Example	Notes about meaning
adjacent to	62	RO:0002220	choroid is adjacent to the sclera	Anatomical descriptor
part of has part	104	BFO:0000050	choroid is part of the uvea	Anatomical descriptor (where the inverse is always true)
characterised by	20	SCDO:0000662	sarcoidosis is characterised by granulomatous histopathology	Disease definition (unidirectional)
investigated by investigation for	84	None applicable	Reiter's investigated by HLA-B27	Investigations included in Royal College of Ophthalmology Consensus document [24] (inverse is always true)
is a	657	None applicable	oral is a route of administration	Defines what something is (unidirectional as it is defining subclass relations)
occurs in	57	BFO:0000066	retinitis occurs in the retina anti-viral is a treatment of viral infection	Descriptor of pathology in relation to anatomy or life-course stage (unidirectional)
treatment of	16	RO:0002606		(unidirectional)

Each concept in OcIMIDo was assigned a unique identifier (e.g. uveitis [OCIMIDO_00213]). We used Protégé’s Pellet reasoner to ensure a coherent and consistent ontology. OcIMIDo had eight top-level classes, three of these included classification [OCIMIDO:00001], complications [OCIMIDO:00003], and therapeutic interventions [OCIMIDO:00004]. The top-level class, classification, included anatomical structure [OCIMIDO:00462], inflammatory disorder [OCIMIDO:00465], and symmetry [OCIMIDO:00306]. The other top-level class, therapeutic interventions [OCIMIDO:00004], contained types of therapy [OCIMIDO:00391], with subclasses of medical therapy [OCIMIDO:00020], and surgical therapy [OCIMIDO:00021]. Supplementary Table 5 summarises OcIMIDo class counts, cross-reference counts and synonym counts, from different sources. Supplementary Table 6 summarises OcIMIDo's frequency of different annotations (labels, comments, cross-references, sources from which data were extracted, synonyms, and sources of synonym extraction), and illustrates these annotations with respect to one disease, Vogt-Koyanagi-Harada disease [OCIMIDO:00108].

### 3.2 Online data extracted from patient support fora

The OV forum contained 2176 posts on 14 March 2019, including 416 primary topic threads, with an average of 5.2 posts (standard deviation 3.9) per thread, ranging from 1 to 16. The UVE forum contained 6855 posts on 6 October 2019, split into 1488 primary topic threads, with an average of 4.6 posts (standard deviation 3.2) per thread, ranging from 0 to 10. Together there were 1904 threads and 9031 forum posts.

### 3.3 Identifying patient preferred synonyms to add to OcIMIDo

The tf-idf analysis on the OV training set (334 threads/1731 posts/6032 words resulted in an additional 208 new terms for inclusion in the ontology (187 synonyms and 21 classes, taking less than 5-min to manually check the list of 653 ranked terms with scores >1.0, and 30 min for all). Many words were considered irrelevant by the domain expert, for example, “time”, “day”, and “hospital”. Examples of words added as synonyms include, “drop”, “steroid”, “humira”, “flare”, “treatment”, and “pressure”.

### 3.4 Validation

First, we repeated the tf-idf analysis on the test set (82 threads/428 posts in 0.6 s). The distribution of a selection of tagged OcIMIDo classes appears very similar in the training and test sets (see Supplementary Fig. 3). For example, 74% of posts in both sets mentioned “uveitis”, 22% and 23% mentioned “corticosteroids” and 27% in both mentioned “methotrexate”. It took 13 min for the domain expert to review the first set of 2581 ranked terms, finding 46 new terms, of which 29 were not “new” synonyms and the other 17 included non-relevant terms. In total, using the tf-idf approach to identify classes and synonyms for inclusion in the ontology from all 2176 posts in OV, took 3 s of computer time, plus approximately 43-min of domain expert time to scan all 8613 terms. Second, tf-idf analysis on the full OV and UVE fora (12 s, identified that the only non-overlapping items were region-specific drug brand names, for example “xibrom”, which is a nonsteroidal anti-inflammatory drug used in North America.

Third, in a timed 60-min exercise, two clinicians, not involved in developing the ontology, independently manually highlighted as many words of relevance to ocular IMID as possible. Clinician one identified 103 unique terms in 376 posts, 54 of these were already included as classes and 30 were synonyms found in the tf-idf on the training set (19 remaining). Clinician two identified 100 unique terms in 69 posts, 37 of these were already classes and 20 were synonyms found in the tf-idf on the training set (43 remaining). Of the combined remaining 62 terms, only 10 terms overlapped. A third expert deemed only 6 additional terms were useful synonyms, these included, “attack”, “bouts”, and “blood shot”. Extrapolating, to explore and extract informative terms from all OV and UVE 9031 posts manually, we estimate it would take 24 h for clinician one, 131 h for clinician two, in comparison to 12.2 s for the tf-idf to run and 2 h for the domain expert to scan through 24,312 ranked terms. This illustrates that tf-idf analysis, with expert curation of a ranked list of items, can be a much faster approach for synonym retrieval when compared to a purely manual curation approach.

### 3.5 The impact of synonym inclusion in ontology class mentions

Adding patient-preferred synonyms increased the number of posts identified to be relevant to the sentiment analysis on account of containing tagged classes. For example, using “methotrexate” with synonyms “mtx”, “mxt” [a common misspelling based on ranking], and “amethopterin” increased the post count by 80%, from 185 to 333; using “adalimumab” with synonym “humira” increased the post count by 3014%, from 7 to 218 (see Supplementary Fig. 4).

### 3.6 Annotating the fora data using OcIMIDo

Exploration of the 9014 posts from the two discussion/support fora provided insight into aspects of inflammatory eye disease and its management of interest or concern to the patients using these two patient support fora (See Table 2 for illustrative selection). Discussion of the subtypes of inflammatory eye disease differed between OV and UVE. For example, anterior uveitis was mentioned by 5.7% (n = 124) OV posts and 8.4% (n = 577) UVE posts (p < 0.001), optic neuritis was mentioned by no posts in OV but 1.0% (n = 70) in UVE, scleritis was mentioned in 0.1% (n = 2) OV posts and 2.7% (n = 184) UVE posts. Discussion of complications was frequent in both fora. For example, cataract was mentioned in 10.0% (n = 218) OV posts and 7.1%(n = 483) UV posts (p < 0.001) and glaucoma was mentioned in 3.7% (n = 81) OV posts and 5.3%(n = 363) UV posts. Systemic diseases were also frequently mentioned. For example, Juvenile idiopathic arthritis was mentioned in 4.5% (n = 97) OV posts, and 1.5% (n = 105) UVE posts, p < 0.001; whilst multiple sclerosis was mentioned in 0.4%(n = 8) OV posts and 1.7% (n = 119) UVE posts, p < 0.001; and inflammatory bowel disease was mentioned in 0.5% (n = 11) OV posts and 2.2% (n = 152) UVE posts, p < 0.001). Posts often included treatments being used or considered, and some differences may result from differences in treatment preferences and availability in the UK and USA. For example, adalimumab was mentioned in 10.0% (n = 218) OV posts and 7.2% (n = 495) UVE posts, p < 0.001; mycophenolate mofetil was mentioned in 1.9% (n = 41) OV posts and 5.2% (n = 356) UVE posts, p < 0.001). Newer biologic therapies, including ustekinumab, tocilizumab, certolizumab, had few mentions in either fora.

**Table 2:** Illustration of the frequency count of a selection of some of the 661 OcIMIDo class items in the posts from the two patient fora. *OR from Multivariable logistic regression model, also adjusted for first post (versus replies to a thread). Otherwise, odds ratios are for single variable analysis. KEY: OV oliviasvision.org; UVE uveitis.org.

OcIMIDo ontology class	All posts (n = 9031)			Most negative + positive 25th posts, Odds of post expressing negative sentiment OR (95% CI), p value	
	OV forum % (n)	UVE forum % (n)	Chi2 p value	TextBlob (n = 4480)	VADER (n = 4557)
**Examples of some clinical phenotypes**					
Scleritis	0.1 (2)	2.7 (184)	p < 0.001	1.9 (1.2–3.2), p = 0.007	1.3 (0.9–1.9), p = 0.207
Anterior uveitis	5.7 (124)	8.4 (577)	p < 0.001	2.3 (1.7–3.1), p < 0.001*	1.3 (1.0–1.5), p = 0.027
Intermediate uveitis	1.8 (38)	5.8 (397)	p < 0.001	1.5 (1.0–2.1), p = 0.041	0.8 (0.6–1.1), p = 0.145
Macular oedema	1.4 (31)	2.1 (144)	p = 0.046	1.3 (0.7–2.4), p = 0.389	0.5 (0.3–0.7), p = 0.001
Posterior uveitis	0.9 (20)	2.6 (179)	p < 0.001	1.4 (0.8–2.3), p = 0.263	0.5 (0.3–0.7), p < 0.001
Panuveitis	1.5 (32)	1.7 (113)	p = 0.565	1.8 (1.0–3.5), p = 0.070	0.6 (0.4–1.0), p = 0.036
Optic neuritis	0 (0)	1.0 (70)	p < 0.001	1.1 (0.5–2.2), p = 0.895	0.9 (0.5–1.7), p = 0.829
**Examples of some associated systemic or neurological diseases**					
Ankylosing spondylitis	0.7 (16)	1.6 (109)	p = 0.003	1.3 (0.6–2.5), p = 0.515	0.8 (0.5–1.3), p = 0.370
Behcet's disease	0.3 (7)	1.3 (92)	p < 0.001	0.7 (0.4–1.4), p = 0.290	0.7 (0.4–1.2), p = 0.214
Inflammatory bowel disease	2.2 (152)	0.5 (11)	p < 0.001	1.3 (0.7–2.3), p = 0.351	1.0 (0.6–1.4), p = 0.844
Birdshot	0.60 (13)	1.01 (69)	p = 0.080	0.8 (0.3–1.7), p = 0.516	0.4 (0.2–0.7), p = 0.001
Juvenile idiopathic arthritis	4.5 (97)	1.5 (105)	p < 0.001	1.7 (1.0–3.0), p = 0.052*	0.6 (0.4–0.9), p = 0.017
Multiple sclerosis	0.4 (8)	1.7 (119)	p < 0.001	2.1 (1.0–4.2), p = 0.049	1.0 (0.6–1.5), p = 0.842
Psoriasis	0.3 (7)	1.7 (113)	p < 0.001	0.9 (0.5–1.9), p = 0.813	1.1 (0.7–1.8), p = 0.688
Rheumatoid arthritis	0.7 (16)	1.1 (78)	p = 0.107	2.8 (1.1–7.2), p = 0.029*	1.2 (0.7–2.1), p = 0.453
Cat scratch disease	1.0 (1)	0.2 (8)	p = 0.356	8.2 (1.0–67.5), p = 0.050*	1.2 (0.5–2.7), p = 0.702
Sarcoidosis	0.1 (1)	0.3 (19)	p = 0.046	2.0 (0.4–10.7), p = 0.437	0.5 (0.1–1.6), p = 0.244
**Examples of some ocular complications of disease or its treatment**					
Cataract	10.0(218)	7.1 (483)	p < 0.001	1.6 (1.2–2.2), p = 0.003*	1.5 (1.1–2.0), p = 0.018
Glaucoma	3.72 (81)	5.3 (363)	p = 0.003	1.8 (1.3–2.5), p = 0.001	1.3 (0.9–1.8), p = 0.231
Epiretinal membrane	0.28 (6)	0.93 (65)	p = 0.002	2.7 (0.9–8.5), p = 0.089	2.4 (0.7–8.0), p = 0.145
Ocular Hypertension	1.19 (26)	0.53 (36)	p = 0.001	2.5 (1.0–6.4), p = 0.063	0.2 (0.1–0.5), p = 0.238
Retinal detachment	0.6 (13)	0.47 (32)	p = 0.451	1.0 (0.3–3.4), p = 0.975	0.2 (0.1–0.5), p = 0.001
**Examples of some treatments**					
Oral prednisolone	6.2 (135)	4.8 (329)	p = 0.010	2.2 (1.5–3.4), p < 0.001*	1.3 (1.0–1.7), p = 0.040
Intravitreal triamcinolone	0.09 (2)	0.64 (44)	p = 0.002	7.9 (1.0–64.4), p = 0.055*	1.1 (0.5–2.4), p = 0.742
Topical steroid	6.0 (130)	3.3 (224)	p < 0.001	2.0 (1.3–3.1), p = 0.002*	1.5 (1.1–2.0), p = 0.014
Methotrexate	15.3 (333)	13.6 (937)	p = 0.056	1.5 (1.2–1.9), p < 0.001*	0.8 (0.7–1.0), p = 0.003
Mycophenolate mofetil	1.88 (41)	5.16 (356)	p < 0.001	1.7 (1.2–2.5), p = 0.004	0.6 (0.4–0.7), p < 0.001
Adalimumab	7.22 (495)	10.02 (218)	p < 0.001	1.5 (1.2–1.9), p = 0.001	0.9 (0.7–1.0), p = 0.131
Bevacizumab	0.23 (5)	1.27 (87)	p < 0.001	1.1 (0.5–2.3), p = 0.888	0.2 (0.1–0.4), p < 0.001
Rituximab	0.09 (2)	1.15 (79)	p < 0.001	0.7 (0.4–1.3), p = 0.247	0.2 (0.1–0.5), p < 0.001
Infliximab	3.81 (83)	5.25 (360)	p = 0.007	1.3 (0.9–1.7), p = 0.118	0.7 (0.6–0.9), p = 0.019
TOTAL	100 (2176)	100 (6855)			

### 3.7 Sentiment analysis with OcIMIDo

**Fig. 2:**
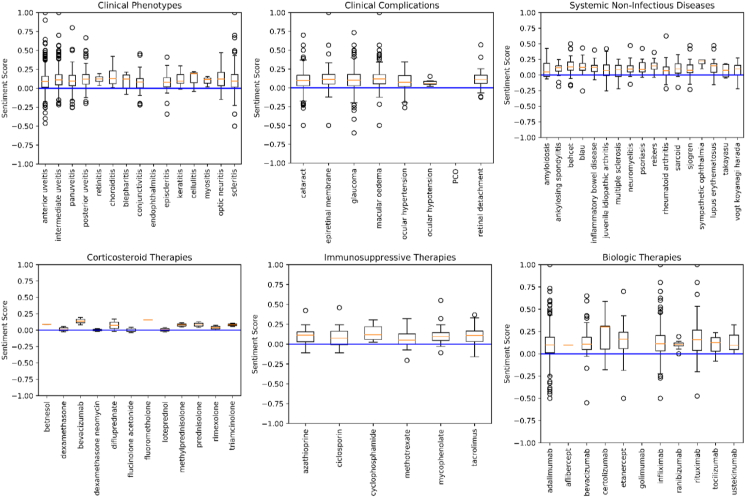
Box plots illustrating spread of natural language sentiment scores with TextBlob for a range of clinical phenotypes, complications, systemic non-infectious diseases, and systemic treatments, combining data from Olivia's Vision and The Ocular Immunology and Uveitis Foundation fora. The scores for each class (including synonyms) ranged from −1.00 (most negative language) to +1.00 (most positive language).

The TextBlob and VADER methods yielded very different median sentiment scores in the full combined dataset, of 0.12 (IQR 0.01–0.24) and 0.77 (0.08–0.93), respectively. The median TextBlob sentiment score was 0.10 (IQR 0 to 0.20) for the 2176 OV posts, and 0.13 (IQR 0.02 to 0.25) for the 6855 UVE posts, with sentiment scores ranging from −1.00 (most negative language) to +1.00 (most positive language). The median VADER sentiment score was 0.69 (IQR 0 to 0.91) for OV posts, and 0.79 (IQR 0.15 to 0.94) for UVE posts. Fig. 2 shows the TextBlob sentiment scores associated with posts mentioning an illustrative selection of different classes (and their synonyms) within the ontology. Using 50% of the data from each forum, after splitting into quartiles of TextBlob and VADER scores, separately, we compared the AUC for the multivariable models of the odds of a post expressing a negative sentiment using either TextBlob (AUC = 0.64, n = 4480 posts) or VADER (AUC = 0.65, n = 4557 posts), see Table 2.

In both multivariable models the odds of a post expressing negative (25th percentile) language sentiment were significantly associated with the first post in a thread, compared to replies (OR 3.3, 95% CI 2.8 to 3.9, p < 0.001 for TextBlob and OR 3.3, 95% CI 2.9 to 3.9, p < 0.001 for VADER), and posts mentioning treatment with oral prednisolone (OR 2.2, 95% CI 1.5–3.4, p < 0.001 for TextBlob, OR 1.3, 95% CI 1.0 to 1.7, p < 0.001 for VADER). In other respects, using data from these sentiment analysis tools, which yielded very different median scores, resulted in models with markedly differing significant associations with OcIMIDO class items. TextBlob median was closest to 0, whilst VADER scores near 1 indicating error towards a ceiling effect, this may be due to the data each were trained on or that VADER's method focuses on individual words and ignoring that word context in which it is used - due to this, we continue to report on sentiment using TextBlob.

## 4 Discussion

We have reported the development and validation of a novel ontology for inflammatory eye disease, OcIMIDo (version 1.2). OcIMIDo organizes 661 classes into high-level concepts of diagnostic subtype, clinical features, classification (anatomy), disease activity, time course, core investigations, therapeutic interventions (and their efficacy and side effects), complications, and functional impacts, with structured knowledge representation. OcIMIDo joins only a small number of other disease area-specific ontologies currently available in general medicine. Notable examples include the @neurIST ontology of intracranial aneurysms [40], and the ACGT Master Ontology of Cancer [41]. Furthermore, we have reported use of an NLP-guided method to identify patient-preferred terms for inclusion in the ontology under class clinical terms as synonyms from a public patient support forum. This addresses an unmet need to better align computational tools with patient and physician-preferred language [1,20].

The OcIMIDo-based annotations confirmed that both of the patient online fora provide a space for patients to discuss a broad range of issues relating to inflammatory eye disease. The differences observed between the fora may relate to differences in the patient groups targeted; UVE is hosted by a USA-based charity and serves patients with a wide range of ocular inflammatory disorders including uveitis, optic neuritis and scleritis; OV is hosted by a UK-based charity catering especially to paediatric uveitis patients.

Sentiment analysis using both tools revealed that first posts were significantly more negative than replies. This provides some insight into the supportive role that online fora play for patients and their carers. The exploratory, multi-variable regression models had reasonable fit (AUC 0.64 and 0.65), given the lack of individual patient data. However, the multiple significant associations between the most negative 25% of post sentiment scores and various ontology classes differed comparing VADER and TextBlob, suggesting that these tools are not measuring sentiment in a concordant way. Manual review of posts and sentiment scores further indicated that sentiment analysis at the level of an entire post provided only limited insight; individual posts frequently contained discussion of a number of disease presentations and treatments (multiple ontology classes), with both positive and negative language sentiments expressed in relation to these.

By illustrating that OcIMIDo, used alongside NLP approaches, facilitates standardised extraction for analysis of real-world unstructured big data in the form of online patient forum posts, we hope to have highlighted how quantitative data can be obtained from this valuable, untapped patient-reported resource. There is increasingly urgent recognition of the need to better understand and integrate the ‘patient voice’ in clinical care pathways, research priority setting, and the development of meaningful outcome measure [1]. We propose that patient support fora could play a particularly helpful role in identifying issues of concern, and advancing patient-centred care for rarer diseases.

### 4.1 Implications

OcIMIDo is freely available to the community as an open source tool, via GitHub (https://github.com/sap218/ocimido). The classes in OcIMIDo were largely uncovered by existing biomedical ontologies (see Supplementary Table 5). Furthermore, OcIMIDo provides an implicit axiomatic structure for data annotated with SNOMED-CT [19], ICD-10 [17], or Read codes [18], as these are included as cross-references in the ontology. This enhances the potential value of OcIMIDo for searching and curating unstructured clinical data. Clinician researchers in other medical specialties, such as rheumatology, gastroenterology, infectious disease, and neurology, may find OcIMIDo a useful resource to use, for advancing the understanding of the inflammatory ocular manifestations of the systemic diseases they manage.

Our study revealed that two commonly used sentiment analysis tools, TextBlob and VADER, yielded very different sentiment scores. Further work is needed to explore the possible reasons for this. Developing OcIMIDo was part of a wider program of work by our group, which includes the development of a semantic framework to fully capture health-related quality of life (QoLo). In future research, our team aims to conduct more advanced applications of OcIMIDo and QoLo together, to improve our understanding of the impacts of inflammatory eye disease and its treatment on patients, and to identify novel content for inclusion in patient reported outcome measures, thereby helping to overcome one of the key barriers to their development.

In a future revision and expansion of OcIMIDo, beyond the currently defined robust relationships, we aim to include an expanded set of classes and axioms relating to evidence-based investigations, risk factors, symptoms, and their inter-relationships, and more extensive cross-references to SNOMED-CT, Read codes and the soon-to-be adopted ICD-11. We will also consider further classifying synonyms as exact (precise alternatives), narrow (more specific), broad (general), or related (associated terms). The value of more descriptive axiomatisation is that it will further enable ontology-based analysis techniques, such as semantic similarity, which can be used to risk stratify patients [42]. OcIMIDo could be further expanded to include additional inflammatory and infectious IMIDs associated with other neuro-ophthalmic, corneal, orbital and adnexal disease presentations. We could add synonyms identified from additional online patient support groups internationally for different ocular and systemic IMIDs. Further potential applications include exploring clinical phenotypes and genetic phenotypes in biobank databases [43], text-mining both open source and EHR data, performing cluster analysis to study underlying relationships in patients with a given disease, and developing ontology-guided predictive algorithms [7].

### 4.2 Limitations

This study has a number of limitations. Firstly, in relation to the ontology development, the Royal College of Ophthalmology consensus document used as the ontology foundation lacked detail on relationships [27]. In addition, a small number of clinical specialists with domain expertise were involved in the development of version 1.2. As we, and other research teams in the community, make use of this free, open access tool, we anticipate that OcIMIDo will be further revised and expanded, with user suggestions and changes being publicly visible and tracked on the repository's “issues” tab. Secondly, as a data resource, unstructured text has multiple limitations. Spell-checking modules in Python do not recognise many terms (e.g. drugs) which we curated as synonyms. In future updates, low-count tf-idf terms, which were misspelled, could potentially be addressed by distance measure techniques. Moreover other synonym curation techniques, such as word2vec, could be considered as they capture semantic similarity in terms of vectors, we did not consider using these techniques as they don't capture the whole document and all terms in terms of rank: the tf-idf and word2vec methods would not be directly comparable - other techniques would be future endeavours. Thirdly, a limitation of using natural language sentiment analysis to illustrate an application of the ontology, was that since the unit of analysis was online posts, extracted retrospectively and without reference to user names, posts may not have been independent (i.e. one patient/carer may have posted/replied on multiple occasions). Additionally labelling the forum is a time-consuming task and there is a lack of publicly labelled patient forum conversation to develop a ML model, so we used pretrained models, in future we would explore developing a trained model. The emergent clinical phenotypes suggested for each forum should therefore be interpreted cautiously, as should the modelled associations between class terms and sentiment scores, which were purely exploratory.

It is important to highlight that ontologies are only part of the solution to extracting structured data from unstructured text. OcIMIDo must be used with other NLP computational tools to annotate text data, and manual review may still play an important role. We have illustrated the simple instantiation of ontology classes in an unstructured dataset, but additional tools will be needed for more nuanced analysis [44]. For example, a given class term, such as ‘methotrexate, might be mentioned in different contexts, such as, ‘history of methotrexate use’, ‘current methotrexate use’, ‘allergy to methotrexate’, or ‘no prior methotrexate use’. Stanford CoreNLP provides context disambiguation tools to help address this by determining information such as bearer (who the mention refers to), temporal status, negation, and uncertainty.

### 4.3 Conclusions

We have demonstrated that the development of an ontology for a specific disease area, enriched with patient-preferred synonyms, provides a potential solution to the challenges arising from the expanding volume of valuable but unstructured patient-reported text data, and the differences between patients' and clinicians' vocabularies and terminologies. Our approach to developing and validating an ontology, using online patient support fora as a data source to permit incorporation of the ‘patient voice’, is readily applicable to other areas of medicine.

## Ethics statement

The University of Birmingham Ethics Committee determined that approval was not required for this study (ERN_20-0047). The research adhered to the tenets of the Declaration of Helsinki.

## Data availability

Yes - all data are fully available without restriction.

The corresponding author confirms that they had full access to all the data in the study and had final responsibility for the decision to submit for publication. Online forum textual data collected is already publicly available - users are able to access without credentials. We have made the ontology available at https://github.com/sap218/ocimido via a creative commons license (CC 3.0).

## Financial disclosure

SCP and GVG acknowledge support from the Medical Research Council (MR/S502431/1) that directly funded this work (https://mrc.ukri.org/).

LTS and GVG also acknowledge support from Horizon 2020 E-Infrastructures (H2020-EINFRA) (731075) (https://ec.europa.eu/programmes/horizon2020/en) in addition to NanoCommons (H2020-EU) (731032) (https://www.nanocommons.eu/).

AK was supported by the Medical Research Council (MR/S003991/1) (https://mrc.ukri.org/).

RMG, GVG, and AKD acknowledge support from the Health Data Research UK (https://www.hdruk.ac.uk/), GVG being directly funded (HDRUK/CFC/01).

GVG acknowledges support from the National Institute for Health Research (https://www.nihr.ac.uk/) Birmingham Experimental Cancer Medicine Centres (https://www.ecmcnetwork.org.uk/), NIHR Birmingham Surgical Reconstruction (https://www.birminghambrc.nihr.ac.uk/) and Microbiology Research Centre (https://srmrc.nihr.ac.uk/), and the NIHR Birmingham Biomedical Research Centre (https://www.uclh.nhs.uk/Research/BRC/Pages/Home.aspx).

TB was part-funded for clinical research by patient charity Olivia’s Vision (http://www.oliviasvision.org/).

The funders had no role in study design, data collection and analysis, decision to publish, or preparation of the manuscript.

## CRediT authorship contribution statement

**Samantha C Pendleton:** Data curation, Methodology, Software, Writing - Original draft preparation. **Karin Slater:** Conceptualization of this study, Methodology, Writing - Original draft preparation. **Andreas Karwath:** Data curation, Methodology, Writing - Original draft preparation. **Rose M Gilbert:** Methodology, Writing - Original draft preparation. **Konrad Pesudovs:** Writing - Original draft preparation. Xiaoxuan Liu: Methodology, Writing - Original draft preparation. **Alastair K Denniston:** Conceptualization of this study, Writing - Original draft preparation. **Georgios V Gkoutos:** Conceptualization of this study, Writing - Original draft preparation. **Tasanee Braithwaite:** Conceptualization of this study, Methodology, Software, Writing - Original draft preparation.

## Declaration of competing interest

The authors have declared that no competing interests exist.

## Appendix A Supplementary data

Multimedia componenet 1 Multimedia componenet 1
