# Supplementary material for: Development and application of the ocular immune-mediated inflammatory diseases ontology enhanced with synonyms from online patient support forum conversation
Source: Comput Biol Med. 2021 Aug;135:104542. doi: 10.1016/j.compbiomed.2021.104542 (PMC8404035; doi:10.1016/j.compbiomed.2021.104542)
Supplement: Multimedia componenet 1 [file mmc1.pdf]

A. Supplementary Tables

Table 3
Summary of the key biomedical ontologies.

Ontology	Description
BFO	The "basic formal ontology" was designed in 2005 for use in supporting information retrieval, analysis and integration in scientific and other domains (9).
DOID	The "human disease ontology" was developed in 2011, this ontology initially included over 8043 terms relating to inherited, developmental, and acquired human disease (10). In 2019 there were over 17930 terms.
HPO	The "human phenotype ontology" was developed in 2008 and included 8000 distinct phenotypic features (11), which have been expanded over time to include ocular symptoms (12). The 2018 HPO release included 1106 terms relating to ocular phenotypes and 968 synonyms related to these terms; 7702 annotations of ocular phenotypes to 2770 rare disorders (13).
ORDO	The "orphanet rare disease ontology" has developed over the past two decades into a formal ontology in 2014. Terms in ORDO correspond to specific rare diseases and their relationships to genes and other features (14). The 2018 version included 1202 rare eye disease-related entries (13). In 2019 there were 14259 classes.
PATO	The "phenotype and trait ontology" was developed in 2002, and includes 2730 classes relating to phenotypes and traits with associated symptoms (6).
RO	The Open Biological and Biomedical Ontologies Foundry "relations ontology" was developed in 2005 with 80 terms as of 2018, and a collection of relations intended for use across a wide variety of biological ontologies (15).
UBERON	The "uber-anatomy ontology" was developed in 2012 and originally consisted of over 6500 classes representing a variety of anatomical entities, including eye anatomy (16).
**Other structured medical vocabularies**	**Description**
ICD-10	The "International Classification of Diseases" version 10 began in 1983 and was first used in 1994. ICD-10 contains codes for diseases, signs and symptoms, abnormal findings, complaints, social circumstances, and external causes of injury or diseases (18). ICD version 11 is due to come into effect in 2022 and is an ontology-based terminology and classification system.
Read Codes	"Read codes" were introduced in the 1980s to provide a structured vocabulary for standardised electronic coding in primary care general practice in the United Kingdom (19). Read codes are not an ontology.
SNOMED-CT	The "Systematized Nomenclature of Medicine - Clinical Terms" was created in 1999, developed by the UK National Health Service (NHS) and released in 2002. It is a systematically organised ontology-based collection of medical terms, synonyms and definitions, used in electronic health records (20).

Table 4
What is an ontology? This table outlines the concepts typically included in a computational ontology.

Concept	Description
IRI and unique identifiers	The concepts in an ontology are represented by Internationalized Resource Identifiers (IRI). IRIs are like unique Uniform Resource Locators (URL) for each concept within the ontology, which locate that concept to a web location. These IRIs are more easily recognised by their unique numeric identifier in the ontology. For example, Vogt-Koyanagi-Harada syndrome has the IRI, http://purl.obolibrary.org/obo/DOID_12297 and the unique identifier [DOID:12297] in the DOID ontology.
Classes	Classes represent the main entities of our domain. Classes can have subclasses to further describe our area of interest. For example, a class, "uveitis" can have "anterior uveitis" and "posterior uveitis" as subclasses.
Relationships	Relationships link classes together to provide a deeper meaning and understanding. Some relationship examples include: "adjacent to", "occurs in", or "part of" (See Table 2 for other relationships).
Axioms	Axioms are formal rules (also known as logical relationships) that are inferred from the ontology structure and are machine readable, allowing deeper understanding of how the classes relate to one another, facilitating semantic interoperability. For example, if "retinitis" is a subclass of "posterior uveitis", and "posterior uveitis" is a subclass of "uveitis", then the ontology infers the axiom that "retinitis" is a subclass of "uveitis".
**Annotations**	
Label	A label is attached to each concept, and is a term that formally represents the domain, [DOID:12297] has the label, Vogt-Koyanagi-Harada syndrome.
Definition	A definition is a comment which describes the concept in more detail, to help the user to understand the concept and its purpose.
Cross-references	A cross-reference links an ontology concept to a concept in another ontology. Many ontology domain content overlap, for example, uveitis has a unique identifier in HPO [HP:0000554], ORDO [ORPHA:98715], and DOID [DOID:13141].
Synonyms	A synonym is a word or phrase that means exactly or nearly the same as a label in the ontology. Commonly synonyms in an ontology are determined by "exact", "related", "broad", or "narrow".

Table 5
Summary of OcIMIDo sources, count of cross-references, and synonyms.

Source	Class count	Cross-reference count	Synonym count
Royal College of Ophthalmology	210	0	9
Human Disease Ontology (DOID)	3	105	0
Human Phenotype Ontology (HPO)	67	158	0
Orphanet Rare Disease Ontology (ORDO)	3	63	0
Phenotype and Trait ontology (PATO)	14	28	0
Uber-anatomy Ontology (UBERON)	61	63	0
Reference Ontology (RO)	0	2	0
Basic Formal Ontology (BFO)	0	2	0
Symptom ontology (SYMP)	0	9	0
Sickle Cell Disease Ontology (SCDO)	0	1	0
International Classification of Disease version 10 (ICD-10)	0	107	0
International Classification of Disease version 9 (ICD-9)	0	25	0
Read Codes	0	170	0
Systemised nomenclature of medical clinical terms (SNOMED-CT)	2	398	0
Olivia's Vision	21	0	187
Expert Opinion	280	0	5
TOTAL	661	1131	201

Table 6
Summary of counts of different annotations within OcIMIDo and illustration using Vogt-Koyanagi-Harada disease [OCIMIDO:00108].
*OWL syntax for annotation.

**Annotation**	**Annotation***	**Counts of all annotations of this type in ocIMIDo**	**One example of annotation in OcIMIDo**
Label	rdfs:label	623	Vogt Koyanagi Harada disease
Definition	rdfs:comment	315	Rare disease that affects several parts of the body, including the eyes, ears, nervous system, and skin
Cross-reference	rdfs:databaseCrossReference	948	DOID:12297<br>ICD10:H208<br>ORPHA:3437<br>readcode:F4423<br>SNOMEDCT:193497004
Source of class	rdfs:extractedFrom	381	Royal College of Ophthalmology Uveitis Clinical Dataset
Synonym	rdfs:hasExactSynonym	191	VKH
Source of synonym	rdfs:synonymExtraction	140	Olivia's Vision Patient Support Forum

B. Supplementary Figures

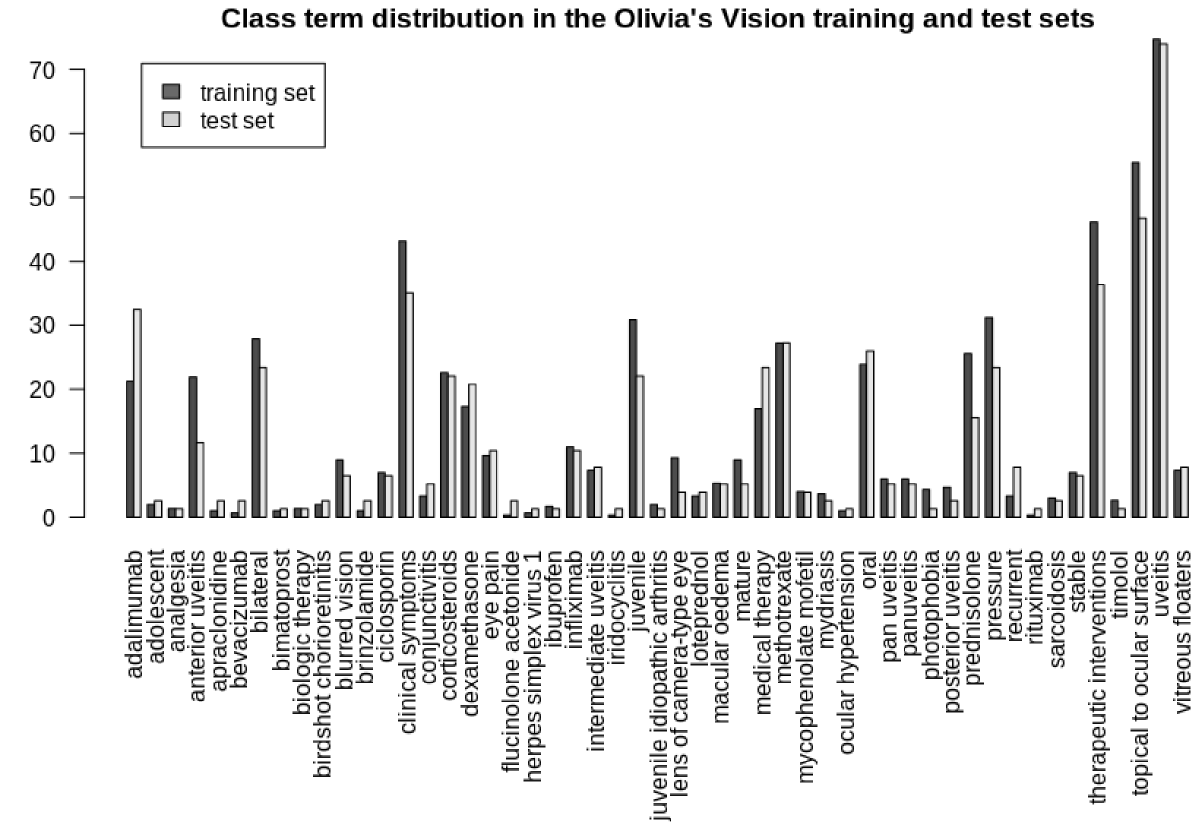

Figure 3: Bar graph illustrating similar class distribution across Olivia's Vision forum training and test sets.

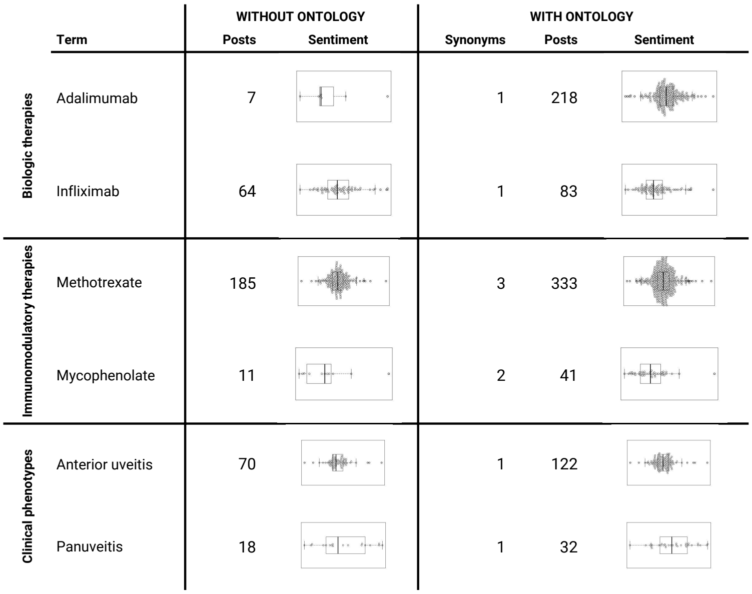

Figure 4: Illustration of synonym inclusion difference for a given class. Showing the amount of available data for analysis increased - for example, adding two synonyms for mycophenolate increased the number of posts mentioning this drug by 30.

CRediT authorship contribution statement

Samantha C Pendleton: Data curation, Methodology, Software, Writing - Original draft preparation. **Luke T Slater:** Conceptualization of this study, Methodology, Writing - Original draft preparation. **Andreas Karwath:** Data curation, Methodology, Writing - Original draft preparation. **Rose M Gilbert:** Methodology, Writing - Original draft preparation. **Konrad Pesudovs:** Writing - Original draft preparation. **Xiaoxuan Liu:** Methodology, Writing - Original draft preparation. **Alastair K Denniston:** Conceptualization of this study, Writing - Original draft preparation. **Georgios V Gkoutos:** Conceptualization of this study, Writing - Original draft preparation. **Tasanee Braithwaite:** Conceptualization of this study, Methodology, Software, Writing - Original draft preparation.

References

[1] S. Dean, J. M. Mathers, M. Calvert, D. G. Kyte, D. Conroy, A. Folkard, S. Southworth, P. I. Murray, and A. K. Denniston, "“the patient is speaking”: discovering the patient voice in ophthalmology," *Br. J. Ophthalmol.*, vol. 101, pp. 700–708, June 2017.

[2] R. Hoehndorf, P. N. Schofield, and G. V. Gkoutos, "The role of ontologies in biological and biomedical research: a functional perspective," *Briefings in Bioinformatics*, vol. 16, pp. 1069–1080, Nov. 2015.

[3] M. A. Haendel, C. G. Chute, and P. N. Robinson, "Classification, ontology, and precision medicine," *N. Engl. J. Med.*, vol. 379, pp. 1452–1462, Oct. 2018.

[4] B. Aldosari, A. Alanazi, and M. Househ, "Pitfalls of ontology in medicine," *Stud. Health Technol. Inform.*, vol. 238, pp. 15–18, 2017.

[5] T. R. Gruber, "Toward principles for the design of ontologies used for knowledge sharing?," *Int. J. Hum. Comput. Stud.*, vol. 43, pp. 907–928, Nov. 1995.

[6] G. V. Gkoutos, P. N. Schofield, and R. Hoehndorf, "The anatomy of phenotype ontologies: principles, properties and applications," *Briefings in Bioinformatics*, vol. 19, pp. 1008–1021, Sept. 2018.
